# The crossroad between tumor and endothelial cells

**DOI:** 10.1007/s10238-024-01490-1

**Published:** 2024-09-26

**Authors:** Domenico Ribatti

**Affiliations:** Department of Translational Biomedicine and Neuroscience, University of Bari Medical School, Università Degli Studi Di Bari, Piazza Giulio Cesare 11, 70125 Bari, Italy

**Keywords:** Endothelial anergy, Endothelium, Inflammation, Metastasis, Tumor growth

## Abstract

Endothelial cells are critical in tumor development, and the specific targeting of endothelial cells offers a potent means to effectively impede angiogenesis and suppress the growth of tumors. Tumor endothelial cells are responsible for the loss of anticancer immunity, the so-called endothelial anergy, i.e., the unresponsiveness of tumor endothelial cells to pro-inflammatory stimulation, not allowing adhesion of immune cells to the endothelium. Endothelial cells downregulate antigen presentation and recruitment of immune cells, contributing to immunosuppression. Targeting endothelial cells may assist in improving the immune effect of immune cells in tumor microenvironment.

## The paradigmatic interactions between leukocytes and endothelial cells during inflammation

Some of the endothelial markers are expressed after activation by inflammatory cytokines. During acute inflammation, the events involving the extravasation of leukocytes from the vascular lumen to the extravascular space include margination and rolling; adhesion and transmigration between endothelial cells; and tissue migration toward a chemotactic stimulus. The selectins mediate the initial rolling interactions of leukocytes with endothelial cells [29]. There are three classes of selectins: L-selectin, expressed on leukocytes, E-selectin, expressed on endothelium, and P-selectin, expressed on platelets and endothelium. P-selectins are stored in secretory granules and released during inflammatory processes. The ligands for selectins are sialylated oligosaccharides bound to mucin-like glycoprotein on target cells. The expression of selectins and their ligands is regulated by cytokines, such as interleukin-1 (IL-1) and tumor necrosis factor alpha (TNFα), produced in response to infection and injury. Leukocytes interact with endothelial E-selectin through CD44 and E-selectin ligand 1 (ESL1) [23]. The binding of leukocytes to E-selectin activates p38 mitogen-activated protein kinase (MAPK)-dependent pathways and α4β7 integrin, favoring leukocyte adhesion to the endothelium [47]. Leukocyte-derived L-selectins and endothelial P-selectin bind to P-selectin glycoprotein ligand 1 (PSLG1) , expressed on leukocytes and endothelial cells [34].

After the rolling, firm adhesion to the surface of the endothelium is mediated by a family of heterodimeric leukocyte surface proteins called integrins. Endothelial cells express ligands for integrins, including vascular cell adhesion molecule-1 (VCAM-1) for the β1 integrin, very late antigen-4 (VLA-4), and intercellular cell adhesion molecule-1 (ICAM-1) for the β2 integrins, lymphocyte function-associated antigen-1 (LFA-1) and macrophage-1 antigen (Mac-1). ICAM-1 and VCAM-1 expressed on endothelial cells facilitate T cell extravasation [56]. ICAM-1 is upregulated at sites of inflammation in response to TNFα and IL-1β [31]. TNFα and transforming growth factor beta (TGFβ) secreted by CD11b^+^ cells activate endothelial cells to secrete chemokine ligand 2 and 7 (CCL2 and CCL7), which recruit natural killer (NK) cells and induce the expression of ICAM-1 and VCAM-1 on endothelial cells and enhance the adhesion of NK cells [58].

High endothelial venules (HEVs) express on their luminal surface ICAMs, VCAMs, and mucosal addressin cell adhesion molecules (MADCAMs), as a ligand for L-selectin, involved in lymphocyte recruitment [17, 42]. The interferon (IFN) gene (STING) stimulator is highly expressed on HEV endothelial cells. Its activation upregulates the expression of ICAM-1 and VCAM-1 on endothelial cells and reprograms tumor vasculature [54]. Moreover, HEVs in tertiary lymphoid organs express podoplanin and secrete CCL21 involved in lymphocyte migration [43].

## Tumor endothelial cells interactions with tumor cells

Tumor endothelial cells are morphologically and phenotypically dysregulated, express specific cell surface markers, and decrease antigen presentation and immune cell recruitment, resulting in tumor immunosuppression [2, 38].

Tumor cells express ligands for E-selectin, leading to rolling and extravasation on endothelial cells expressing E-selectin. Colon carcinoma cells express HCELL, a ligand for E-selectin [7]. Bone metastatic prostate carcinoma cell lines express P-selectin glycoprotein ligand-1 (PSGL-1) and the E-selectin ligand-1 (ESL-1) [12]. Carcinoembryonic antigen (CEA) acts as a mediator for the rolling of colon carcinoma cells on E-and L-selectins [49]. Moreover, selectins on endothelial cells and corresponding T cell receptors are involved in the interaction of   chimeric antigen receptor (CAR)-T cells with tumor cells [33].

Endothelin-1 (ET-1) is involved in reducing the expression of ICAM-1 on tumor endothelial cells and of tumor-infiltrating leukocytes [6]. Lymphocytes roll through VCAM-1 interaction with VLA-4 [3]. Epstein–Barr virus (EBV)^+^ NK lymphoma cell lines release TNFα responsible for an increase of the expression of ICAM-1 and VCAM-1 on endothelial cells and enhanced adhesion of NK cells [27].

Tumor cells activate Notch1 signaling activity in tumor endothelial cells facilitating tumor cell transmigration, intravasation, and metastasis [57]. Tumor endothelial cells express RAE1ɛ, which activates NKG2D causing its internalization from NK cells surface desensitizing NK antitumor responses [50].

Human endothelial cells express on their surface major histocompatibility complex (MHC) class I and II molecules [41]. Tumor endothelial cells downregulate genes involved in MHC expression and impede their antigen-presenting functions [19]. MHC class II antigen presentation by tumor lymphatic endothelial cells promotes intratumoral Treg suppressive function [18]. IFN gamma (IFNγ) activated dermal tumor lymphatic endothelial cells inhibit cytotoxic T cells accumulation in melanoma [28]. Tumor-associated macrophages (TAMs) express high levels of podoplanin promoting their attachment to lymphatic endothelial cells and lymphoinvasion [4]. Galectin-1 (Gal-1) expression by tumor endothelial cells inhibits T cell trans endothelial migration induced by prostate cancer cells [22].

Tumor endothelial cells express Fas ligand (FasL) involved in T cell apoptosis reducing  CD8^+^ T cells in tumors. [35], express and upregulate indoleamine 2,3 dioxygenase 1 (IDO1), an enzyme reducing T cell proliferation, and IDO1 inhibition enhances the effect of CD40 stimulating immunotherapy [16].

Nitric oxide (NO) affects leukocyte recruitment reducing tumor endothelial cell expression of adhesion molecules (E-selectin, VCAM-1, and ICAM-1) and pro-inflammatory cytokines [10]. Endothelial cells downregulate Slit2, a tumor-suppressive angiocrine factor inhibited by ephrin 2 (Eph2), facilitating tumor cell proliferation and motility [5]. The angiocrine factor biglycan is expressed by tumor endothelial cells of highly metastatic tumors [32].

## Endothelial cell anergy

Tumor endothelial cells are responsible for the loss of anticancer immunity, the so-called endothelial anergy, i.e., the unresponsiveness of tumor endothelial cells to pro-inflammatory stimulation, not allowing adhesion of immune cells to the endothelium [11, 20]. Endothelial cells cannot be triggered by inflammation and cannot activate endothelial adhesion molecules [26].

Vascular endothelial growth factor (VEGF) and fibroblast growth factor 2 (FGF2) secreted by tumor cells induce tumor endothelial cells anergy by inhibiting the upregulation of ICAM-1, VCAM-1, and selectins on tumor endothelial cells under inflammatory stimulation [21]. VEGF blockade restores adhesion molecule expression and interactions between leukocytes and endothelial cells [14]. Tumor-infiltrating leukocytes are markedly increased following VEGF inhibition [15, 46]. VEGF suppresses T cell functions through the induction of FasL  expression on endothelial cells and of apoptosis of infiltrating CD8^+^ T cells [35].

Oral squamous cell carcinoma tumor cells release VEGF, inducing further release of VEGF by tumor endothelial cells and inhibiting T cells [36]. Tumor cells of Lewis lung carcinoma secrete VEGF, inducing prostaglandin E2 (PGE2) production by tumor endothelial cells, and suppressing T cell function [37]. In hepatocellular carcinoma, tumor endothelial cells induce tumor-infiltrating T cell exhaustion by expressing glycoprotein nonmetastatic melanoma protein B (GPNMB) [44].

## Immune checkpoint inhibitors

Tumor endothelial cells express programmed death-ligand1 (PD-L1) and PD-L2, which interact with PD-1 expressed on CD8^+^ T cell surface cells and inhibit their antitumor activity. Pro-inflammatory cytokines, such as IFNγ and TNFα, enhance PD-L1 upregulation on endothelial cells. High expression of PD-L1 on tumor endothelial cells reduces CD8^+^ T cell infiltration and increases the aggregate of forkhead box P3 (FoxP3)^+^ Tregs in the tumor mass; anlotinib downregulates PD-L1 expression on tumor endothelial cells [30]. FoxP3^+^ Tregs suppress immune response to tumors resulting in a poor prognosis. PD-L1 deficiency in tumor endothelial cells reduces their ability to induce apoptosis in tumor-infiltrating CD8^+^ T cells, inhibiting tumor development [48]. Tumor endothelial cells induce immunosuppressive CD4^+^ T cells through IL-10 and TGFβ, contributing to tumor evasion [48]. The expression of T cell immunoglobulin and mucin domain 3 (TIM3) in lymphoma endothelial cells inhibits CD4^+^ T cell activation and Th1 polarization, favoring immunoevasion [24]. Overexpression of TIM3 in breast cancer tumor cells stimulates the release of VEGF and angiogenesis [8]. Tumor endothelial cells express the B7-H3 protein, an immune checkpoint protein involved in progression of ovarian and renal cell carcinomas [9, 60].

VEGFA promotes the expression of PD-1, cytotoxic T lymphocyte-associated antigen 4 (CTLA-4), TIM3, and lymphocyte activation gene 3 protein [53].

## Therapeutic correlates

The combination of ipilimumab and bevacizumab induces the expression of E-selectin, ICAM-1, and VCAM-1 on tumor endothelial cells and the adhesion of activated T cells to tumor endothelial cells [59]. CD40/CD40L binding induces leukocyte adhesion to endothelial cells through the E-selectin binding, and inhibits endothelial cell migration [51]. Rolling of leukocytes on HEV is mediated by L-selectin and their ligands, collectively known as peripheral lymph node addressins. HEVs express CCL-21, which activates the LFA-1 [1]. Treatment with an agonist of CD137 (expressed on tumor endothelial cells) monoclonal antibody increases the expression of ICAM-1, VCAM-1, and E-selectin on tumor endothelial cell surface promoting CD8^+^ T cells recruitment [40].

Dual angiopoietin-2 (Ang-2) and VEGF blockade induce a higher expression of VCAM-1 on tumor endothelial cells, allowing the accumulation of antitumor T cells [45]. Combination of sunitinib and anti-CD40 monoclonal antibody treatment increases the expression of ICAM-1 and VCAM-1 on tumor endothelial cells, enhancing intratumoral infiltration of CD8^+^ cytotoxic T cells [52]. CCL-2, CCL-7, ICAM-1, and VCAM-1 may act as targets to enhance NK infiltration in tumors [58].

Anti-angiogenic agents may overcome tumor endothelial cells anergy and improve immunotherapy efficacy [39]. Sunitinib, sorafenib, and angiostatic peptides increase tumor-infiltrating leukocyte infiltration [13, 25,46]. Atezolizumab in combination with bevacizumab enhances migration of antigen-specific T cells in metastatic renal cell carcinoma [55]. Pharmacological inhibition of VEGFA, IL-10, and PGE2 results in lower FasL expression on endothelial cells and higher CD8^+^ T cell infiltration [35].

## Data Availability

No datasets were generated or analyzed during the current study.

## References

[1] Aird WC. Phenotypic heterogeneity of the endothelium: I. Structure, function, and mechanisms. Circ Res. 2007;100:158–73. 17272818 10.1161/01.RES.0000255691.76142.4a

[2] Amersfoort J, Eelen G, Carmeliet P. Immunomodulation by endothelial cells - partnering up with the immune system? Nat Rev Immunol. 2022;22:576–88. 35288707 10.1038/s41577-022-00694-4 PMC8920067

[3] Berlin C, Bargatze RF, Campbell JJ, vonAndrian UH, Szabo MC, Hasslen SR, Nelson RD, Berg EL, Erlandsen SL, Butche EC. α4-integrins mediate lymphocyte attachment and rolling under physiologic flow. Cell. 1995;80:413–22. 7532110 10.1016/0092-8674(95)90491-3

[4] Bieniasz-Krzywiec P, Martín-Pérez R, Ehling M, García-Caballero M, Pinioti S, Pretto S, Kroes R, Aldeni C, Di Matteo M, Prenen H, Tribulatti MV, Campetella O, Smeets A, Noel A, Floris G, Van Ginderachter JA, Mazzone M. Podoplanin-expressing macrophages promote lymphangiogenesis and lymphoinvasion in breast cancer. Cell Metab. 2019;30:917–36. 31447322 10.1016/j.cmet.2019.07.015 PMC7616630

[5] Brantley-Sieders DM, Dunaway CM, Rao M, Short S, Hwang Y, Gao Y, Li D, Jiang A, Shyr Y, Wu JY, Chen J. Angiocrine factors modulate tumor proliferation and motility through EphA2 repression of Slit2 tumor suppressor function in endothelium. Cancer Res. 2011;71:976–87. 21148069 10.1158/0008-5472.CAN-10-3396 PMC3032824

[6] Buckanovich RJ, Facciabene A, Kim S, Benencia F, Sasaroli D, Balint K, Katsaros D, O’Brien-Jenkins A, Gimotty PA, Coukoset G. Endothelin B receptor mediates the endothelial barrier to T cell homing to tumors and disables immune therapy. Nat Med. 2008;14:28–36. 18157142 10.1038/nm1699

[7] Burdick MM, Chu JT, Godar S, Sackstein R. HCELL is the major E and L-selectin ligand expressed on LS174T colon carcinoma cells. J Biol Chem. 2006;281:13899–905. 16565092 10.1074/jbc.M513617200

[8] Cong Y, Cui Y, Zhu S, Cao J, Zou H, Martin TA, Qiao G, Jiang W, Yu Z. Tim-3 promotes cell aggressiveness and paclitaxel resistance through NF-κB/STAT3 signalling pathway in breast cancer cells. Chin J Cancer Res. 2020;32:564–79. 33223752 10.21147/j.issn.1000-9604.2020.05.02 PMC7666787

[9] Crispen PL, Sheinin Y, Roth TJ, Lohse CM, Kuntz SM, Frigola X, Thompson RH, Boorjian SA, Dong H, Leibovich BC, Blute ML, Kwon ED. Tumor cell and tumor vasculature expression of B7–H3 predict survival in clear cell renal cell carcinoma. Clin Cancer Res. 2008;14:5150–7. 18694993 10.1158/1078-0432.CCR-08-0536 PMC2789387

[10] De Caterina R, Libby P, Peng HB, Thannickal VJ, Rajavashisth TB, Gimbrone MA, Shin WS, Liao JK. Nitric oxide decreases cytokine-induced endothelial activation. Nitric oxide selectively reduces endothelial expression of adhesion molecules and proinflammatory cytokines. J Clin Invest. 1995;96:60–8. 7542286 10.1172/JCI118074 PMC185173

[11] De Sanctis F, Ugel S, Facciponte J, Facciabene A. The dark side of tumor-associated endothelial cells. Semin Immunol. 2018;35:35–47. 29490888 10.1016/j.smim.2018.02.002

[12] Dimitroff CJ, Descheny L, Trujillo N, Kim R, Nguyen V, Huang W, Pienta KJ, Kutok JL, Rubin MA. Identification of leukocyte E selectin ligands, P-selectin glycoprotein ligand-1 and E-selectin ligand-1, on human metastatic prostate tumor cells. Cancer Res. 2005;65:5750–60. 15994950 10.1158/0008-5472.CAN-04-4653 PMC1472661

[13] Dings RP, Vang KB, Castermans K, Popescu F, Zhang Y, Oude Egbrink MG, Mescher MF, Farrar MA, Griffioen AW, Mayo KH. Enhancement of T-cell mediated antitumor response: angiostatic adjuvant to immunotherapy against cancer. Clin Cancer Res. 2011;17:3134–45. 21252159 10.1158/1078-0432.CCR-10-2443 PMC4242153

[14] Dirkx AE, Oude Egbrink MG, Kuijpers MJ, van der Niet ST, Heijnen VV, Boumater Steege JC, Wagstaff J, Griffioen AW. Tumor angiogenesis modulates leukocyte-vessel wall interactions in vivo by reducing endothelial adhesion molecule expression. Cancer Res. 2003;63:2322–9. 12727857

[15] Dirkx AE, Oude Egbrink MG, Castermans K, van der Schaft DW, Thijssen VL, Dings RP, Kwee L, Mayo KH, Wagstaff J, Bouma-ter Steege JC, Griffioen AW. Anti-angiogenesis therapy can overcome endothelial cell anergy and promote leukocyte-endothelium interactions and infiltration in tumors. FASEB J. 2006;20:621–30. 16581970 10.1096/fj.05-4493com

[16] Georgina M, Ramachandran M, Tuit S, Núñez NG, Karampatzakis A, Fotaki G, van Hooren L, Huang H, Lugano R, Ulas T, Kaunisto A, Holland EC, Ellmark P, Mangsbo SM, Schultze J, Essand M, Tugues S, Dimberg A. Tumor endothelial cell up-regulation of IDO1 is an immunosuppressive feed-back mechanism that reduces the response to CD40-stimulating immunotherapy. Oncoimmunology. 2020;9:1730538. 32231867 10.1080/2162402X.2020.1730538 PMC7094447

[17] Girard JP, Springer TA. High endothelial venules (HEVs): specialized endothelium for lymphocyte migration. Immunol Today. 1995;16:449–57. 7546210 10.1016/0167-5699(95)80023-9

[18] Gkountidi AO, Garnier L, Dubrot J, Angelillo J, Harlé G, Brighouse D, Wrobel LJ, Pick R, Scheiermann C, Swartz MA, Hugues S. MHC Class II antigen presentation by lymphatic endothelial cells in tumors promotes intratumoral regulatory T cell-suppressive functions. Cancer Immunol Res. 2021;9:748–64. 33952631 10.1158/2326-6066.CIR-20-0784 PMC11095080

[19] Goveia J, Rohlenova K, Taverna F, Treps L, Conradi LC, Pircher A, Geldhof V, de Rooij LPMH, Kalucka J, Sokol L, García-Caballero M, Zheng Y, Qian J, Teuwen LA, Khan S, Boeckx B, Wauters E, Decaluwé H, De Leyn P, Vansteenkiste J, Weynand B, Sagaert X, Verbeken E, Wolthuis A, Topal B, Everaerts W, Bohnenberger H, Emmert A, Panovska D, FDe Smet F, Staal FJT, Mclaughlin RJ, Impens F, Lagani V, Vinckier S, Mazzone M, Schoonjans L, Dewerchin M, Eelen G, Karakach TK, Yang H, Wang J, Bolund L, Lin L, Thienpont B, Li X, Lambrechts D, Luo Y, Carmeliet P,. An integrated gene expression landscape profiling approach to identify lung tumor endothelial cell heterogeneity and angiogenic Candidates. Cancer Cell. 2020;37:21–36. 31935371 10.1016/j.ccell.2019.12.001

[20] Griffioen A, Dame CA, Blijham GH, Groenewegen G. Tumor angiogenesis is accompanied by a decreased inflammatory response of tumor-associated endothelium. Blood. 1996;88:667–73. 8695814

[21] Griffioen AW, Damen CA, Mayo KH, Barendsz-Janson AF, Martinotti S, Blijham GH, Groenewegen G. Angiogenesis inhibitors overcome tumor induced endothelial cell anergy. Int J Cancer. 1999;80:315–9. 9935216 10.1002/(sici)1097-0215(19990118)80:2<315::aid-ijc23>3.0.co;2-l

[22] He J, Baum LG. Endothelial cell expression of galectin-1 induced by prostate cancer cells inhibits T-cell transendothelial migration. Lab Invest. 2006;86:578–90. 16607379 10.1038/labinvest.3700420

[23] Hidalgo A, Peired AJ, Wild M, Vestweber D, Frenette PS. Complete identification of E-selectin ligands on neutrophils reveals distinct functions of PSGL-1, ESL-1, and CD44. Immunity. 2007;26:477–89. 17442598 10.1016/j.immuni.2007.03.011 PMC4080624

[24] Huang X, Bai X, Cao Y, Wu J, Huang M, Tang D, Tao S, Zhu T, Liu Y, Yang Y, Zhou X, Zhao Y, Wu M, Wei J, Wang D, Xu G, Wang S, Ma D, Zhou J. Lymphoma endothelium preferentially expresses Tim-3 and facilitates the progression of lymphoma by mediating immune evasion. J Exp Med. 2010;207:505–20. 20176801 10.1084/jem.20090397 PMC2839144

[25] Huang Y, Yuan J, Righi E, Kamoun WS, Ancukiewicz M, Nezivar J, Santosuosso M, Martin JD, Martin MR, Vianello F, Leblanc P, Munn LL, Huang P, Duda DG, Fukumura D, Jain RK, Poznansky MC. Vascular normalizing doses of antiangiogenic treatment reprogram the immunosuppressive tumor microenvironment and enhance immunotherapy. Proc Natl Acad Sci USA. 2012;109:17561–6. 23045683 10.1073/pnas.1215397109 PMC3491458

[26] Huinen ZR, Huijbers EJM, van Beijnum JR, Nowak-Sliwinska P, Griffioen AW. Antiangiogenic agents-overcoming tumour endothelial cell anergy and improving immunotherapy outcomes. Nat Rev Clin Oncol. 2021;18(8):527–40. 33833434 10.1038/s41571-021-00496-y

[27] Kanno H, Watabe D, Shimizu N, Sawai T. Adhesion of Epstein-Barr virus-positive natural killer cell lines to cultured endothelial cells stimulated with inflammatory cytokines. Clin Exp Immunol. 2008;151:519–27. 18190605 10.1111/j.1365-2249.2007.03584.x PMC2276960

[28] Lane RS, Femel J, Breazeale AP, Loo CP, Thibault G, Kaempf A, Mori M, Tsujikawa T, Chang YH, Lund AW. IFNγ-activated dermal lymphatic vessels inhibit cytotoxic T cells in melanoma and inflamed skin. J Exp Med. 2018;215:3057–74. 30381467 10.1084/jem.20180654 PMC6279400

[29] Ley K, Laudanna C, Cybulsky MI, Nourshargh S. Getting to the site of inflammation: the leukocyte adhesion cascade updated. Nat Rev Immunol. 2007;7:678–89. 17717539 10.1038/nri2156

[30] Liu S, Qin T, Liu Z, Wang J, Jia Y, Feng Y, Gao Y, Li K. Anlotinib alters tumor immune microenvironment by downregulating PD-L1 expression on vascular endothelial cells. Cell Death Dis. 2020;11:309. 32366856 10.1038/s41419-020-2511-3 PMC7198575

[31] Luo BH, Carman CV, Springer TA. Structural basis of integrin regulation and signaling. Annu Rev Immunol. 2007;25:619–47. 17201681 10.1146/annurev.immunol.25.022106.141618 PMC1952532

[32] Maishi N, Ohba Y, Akiyama K, Ohga N, Hamada J, Nagao-Kitamoto H, Alam MT, Yamamoto K, Kawamoto T, Inoue N, Taketomi A, Shindoh M, Hida Y, Hida K. Tumour endothelial cells in high metastatic tumours promote metastasis via epigenetic dysregulation of biglycan. Sci Rep. 2016;6:28039. 27295191 10.1038/srep28039 PMC4904795

[33] Martinez M, Moon EK. CAR T cells for solid tumors: new strategies for finding, infiltrating, and surviving in the tumor microenvironment. Front Immunol. 2019;10:128. 30804938 10.3389/fimmu.2019.00128 PMC6370640

[34] Martins PDC, Garcia-Vallejo JJ, Van Thienen JV, Fernandez-Borja M, van Gils JM, Beckers C, Horrevoets AJ, Hordijk PL, Zwaginga JJ. P-selectin glycoprotein ligand- 1 is expressed on endothelial cells and mediates monocyte adhesion to activated endothelium. Arterioscler Thromb Vasc Biol. 2007;27:1023–9. 17322099 10.1161/ATVBAHA.107.140442

[35] Motz GT, Santoro SP, Wang LP, Garrabrant T, Lastra RR, Hagemann IS, Lal P, Feldman MD, Benencia F, Coukos G. Tumor endothelium FasL establishes a selective immune barrier promoting tolerance in tumors. Nat Med. 2014;20:607–15. 24793239 10.1038/nm.3541 PMC4060245

[36] Mulligan JK, Day TA, Gillespie MB, Rosenzweig SA, Young MR. Secretion of vascular endothelial growth factor by oral squamous cell carcinoma cells skew endothelial cells to suppress T-cell functions. Hum Immunol. 2009;70:375–82. 19480853 10.1016/j.humimm.2009.01.014 PMC2746465

[37] Mulligan JK, Rosenzweig SA, Young MR. Tumor secretion of VEGF induces endothelial cells to suppress T cell functions through the production of PGE2. J Immunother. 2010;33:126–35. 20145550 10.1097/CJI.0b013e3181b91c9c PMC3333835

[38] Nagl L, Horvath L, Pircher A, Wolf D. Tumor endothelial cells (Tecs) as potential immune directors of the tumor microenvironment – new findings and future perspectives. Front Cell Dev Biol. 2020;8:766. 32974337 10.3389/fcell.2020.00766 PMC7466447

[39] Nowak-Sliwinska P, van Beijnu JR, Griffioen CJ, Huinen ZR, Sopesens NG, Schulz R, Jenkins SV, Dings RPM, Groenendijk FH, Huijbers EJM, Thijssen VLJL, Jonasch E, Vyth-Dreese FA, Jordanova ES, Bex A, Bernards R, de Gruijl TD, Griffioen AW. Proinflammatory activity of VEGF-targeted treatment through reversal of tumor endothelial cell anergy. Angiogenesis. 2023;26:279–93. 36459240 10.1007/s10456-022-09863-4 PMC10119234

[40] Palazon A, Teijeira A, Martinez-Forero I, Hervas-Stubbs S, Roncal C, Penuelas I, Juan Dubrot J, Morales-Kastresana A, Pérez-Gracia JL, Ochoa MC, Ochoa-Callejero L, Martínez A, Luque A, Dinchuk J, Rouzaut A, Jure-Kunkel M, Melero I. Agonist anti-cd137 mab act on tumor endothelial cells to enhance recruitment of activated T lymphocytes. Cancer Res. 2011;71:801–11. 21266358 10.1158/0008-5472.CAN-10-1733

[41] Pober JS, Tellides G. Participation of blood vessel cells in human adaptive immune responses. Trends Immunol. 2012;33:49–57. 22030237 10.1016/j.it.2011.09.006 PMC3253953

[42] Rosen SD. Endothelial ligands for L-selectin: from lymphocyte recirculation to allograft rejection. Am J Pathol. 1999;155:1013–20. 10514381 10.1016/S0002-9440(10)65201-7 PMC1867022

[43] Ruddle NH. High endothelial venules and lymphatic vessels in tertiary lymphoid organs: characteristics functions, and regulation. Front Immunol. 2016;7:491. 10.3389/fimmu.2016.00491. 27881983 10.3389/fimmu.2016.00491 PMC5101196

[44] Sakano Y, Noda T, Kobayashi S, Sasaki K, Iwagami Y, Yamada D, Tomimaru Y, Akita H, Gotoh K, Takahashi H, Asaoka T, Tanemura M, Wada H, Doki Y, Eguchi H. Tumor endothelial cell-induced CD8+ T-cell exhaustion via GPNMB in hepatocellular carcinoma. Cancer Sci. 2022;113:1625–38. 35289033 10.1111/cas.15331 PMC9128167

[45] Schmittnaegel M, Rigamonti N, Kadioglu E, Cassará A, Wyser Rmili C, Kiialainen A, Kienast Y, Mueller HJ, Ooi CH, Laoui D, De Palma M. Dual angiopoietin-2 and VEGFA inhibition elicits antitumor immunity that is enhanced by PD-1 checkpoint blockade. Sci Transl Med. 2017;9(385):eaak9670. 28404865 10.1126/scitranslmed.aak9670

[46] Shrimali RK, Yu Z, Theoret MR, Chinnasamy D, Restifo NP, Rosenberg SA. Antiangiogenic agents can increase lymphocyte infiltration into tumor and enhance the effectiveness of adoptive immunotherapy of cancer. Cancer Res. 2010;70:6171–80. 20631075 10.1158/0008-5472.CAN-10-0153 PMC2912959

[47] Simon SI, Hu Y, Vestweber D, Smith CW. Neutrophil tethering on E-selectin activates β2 integrin binding to ICAM-1 through a mitogen-activated protein kinase signal transduction pathway. J Immunol. 2000;164:4348–58. 10754335 10.4049/jimmunol.164.8.4348

[48] Taguchi K, Onoe T, Yoshida T, Yamashita Y, Tanaka Y, Ohdan H. Tumor endothelial cell-mediated antigen-specific T-cell suppression via the PD-1/PD-L1 pathway. Mol Cancer Res. 2020;18:1427–40. 32527950 10.1158/1541-7786.MCR-19-0897

[49] Thomas SN, Zhu F, Schnaar RL, Alves CS, Konstantopoulos K. Carcinoembryonic antigen and CD44 variant isoforms cooperate to mediate colon carcinoma cell adhesion to E- and L-selectin in shear flow. J Biol Chem. 2008;283:15647–55. 18375392 10.1074/jbc.M800543200 PMC2414264

[50] Thompson TW, Kim AB, Li PJ, Wang J, Jackson BT, Huang KTH, Zhang L, Raulet DH. Endothelial cells express NKG2D ligands and desensitize antitumor NK responses. Elife. 2017;6:e30881. 10.7554/eLife.30881. 29231815 10.7554/eLife.30881 PMC5792093

[51] Urban D, Thanabalasingam U, Stibenz D, Kaufmann J, Meyborg H, Fleck E, Gräfe M, Stawowy P. CD40/CD40L interaction induces E-selectin dependent leukocyte adhesion to human endothelial cells and inhibits endothelial cell migration. Biochem Biophys Res Commun. 2011;404:448–52. 21138731 10.1016/j.bbrc.2010.11.142

[52] van Hooren L, Georganaki M, Huang H, Mangsbo SM, Dimberg A. Sunitinib enhances the antitumor responses of agonistic CD40-antibody by reducing MDSCs and synergistically improving endothelial activation and T-cell recruitment. Oncotarget. 2016;7:50277–89. 27385210 10.18632/oncotarget.10364 PMC5226582

[53] Voron T, Colussi O, Marcheteau E, Pernot S, Nizard M, Pointet AL, Latreche S, Bergaya S, Benhamouda N, Tanchot C, Stockmann C, Combe P, Berger A, Zinzindohoue F, Yagita H, Tartour E, Taieb J, Terme M. VEGF-A modulates expression of inhibitory checkpoints on CD8+ T cells in tumors. J Exp Med. 2015;212:139–48. 25601652 10.1084/jem.20140559 PMC4322048

[54] Yang H, Lee WS, Kong SJ, Kim CG, Kim JH, Chang SK, Kim S, Kim G, Chon HJ, Kim C. STING activation reprograms tumor vasculatures and synergizes with VEGFR2 blockade. J Clin Invest. 2019;130:4350–64. 10.1172/JCI125413 PMC6763266 31343989

[55] Wallin JJ, Bendell JC, Funke R, Sznol M, Korski K, Jones S, Hernandez G, Mier J, He X, Hodi FS, Denker M, Leveque V, Cañamero M, Babitski G, Koeppen H, Ziai J, Sharma N, Gaire F, Chen DS, Waterkamp D, Hegde PS, McDermott DF. Atezolizumab in combination with bevacizumab enhances migration of antigen-specific T-cells in metastatic renal cell carcinoma. Nat Commun. 2016;7:12624. 27571927 10.1038/ncomms12624 PMC5013615

[56] Wettschurek N, Strilic B, Offermann S. Passing the vascular barrier: endothelial signaling processes controlling extravasation. Physiol Rev. 2019;99:1467–525. 31140373 10.1152/physrev.00037.2018

[57] Wieland E, Rodriguez-Vita J, Liebler SS, Mogler C, Moll I, Herberich SE, Espinet E, Herpel E, Menuchin A, Chang-Claude J, Hoffmeister M, Gebhardt C, Brenner H, Trumpp A, Siebel CW, Hecker M, Utikal J, Sprinzak D, Fischer A. Endothelial Notch1 activity facilitates metastasis. Cancer Cell. 2017;31:355–67. 28238683 10.1016/j.ccell.2017.01.007

[58] Won Jun H, Kyung Lee H, Ho Na I, Jeong Lee S, Kim K, Park G, Kim HS, Son DJ, Kim Y, Jhong JT, Han SB. The role of CCL2, CCL7, ICAM-1, and VCAM-1 in interaction of endothelial cells and natural killer cells. Int Immunopharmacol. 2022;113: 109332. 36274485 10.1016/j.intimp.2022.109332

[59] Wu X, Giobbie-Hurder A, Liao X, Lawrence D, McDermott D, Zhou J, Rodig S, Hodi FS. VEGF neutralization plus CTLA-4 blockade alters soluble and cellular factors associated with enhancing lymphocyte infiltration and humoral recognition in melanoma. Cancer Immunol Res. 2016;4:858–68. 27549123 10.1158/2326-6066.CIR-16-0084 PMC5050160

[60] Zang X, Sullivan PS, Soslow RA, Waitz R, Reuter VE, Wilton A, Thaler HT, Arul M, Slovin SF, Wei J, Spriggs DR, Dupont J, Allison JP. Tumor associated endothelial expression of B7–H3 predicts survival in ovarian carcinomas. Mod Pathol. 2010;23:1104–12. 20495537 10.1038/modpathol.2010.95 PMC2976590

